# Fluorescence spectral diagnosis of malaria – a preliminary study

**DOI:** 10.1186/s13000-014-0182-z

**Published:** 2014-10-17

**Authors:** Vadivel Masilamani, Sandhanasamy Devanesan, Mani Ravikumar, Kantharaj Perinbam, Mohamad Saleh AlSalhi, Saradh Prasad, Siddanna Palled, Kadirampatti Mani Ganesh, Abbas Habeeb Alsaeed

**Affiliations:** Department of Physics and Astronomy, College of Science, King Saud University, P.O Box 2455, Riyadh, Kingdom of Saudi Arabia; Research Chair for Laser Diagnosis of Cancers, King Saud University, Riyadh, Kingdom of Saudi Arabia; PG and Research Department of Plant Biology and Biotechnology, Govt Arts College for Men, Nandanam, Chennai, India; Radiation Oncology Department, KMIO, Bangalore, India; College of Medical Sciences, King Saud University, Riyadh, Saudi Arabia

**Keywords:** Malaria diagnosis, Fluorescent biomarkers, Spectral diagnosis, Hemoglobin damage, Plasmodium falciparum

## Abstract

**Background:**

Malaria is the most common disease transmitted by the bite by an infected female anopheles mosquito and caused by the plasmodium parasite. It is mostly prevalent in subtropical regions receiving abundant rain and supporting copious mosquito breeding. This disease is generally detected by the microscopic examination of blood films or antigen based rapid diagnostic test. Only occasionally the parasite DNA is detected using polymerase chain reaction in certain advanced, expensive laboratories.

**Methods:**

An innovative spectral detection method based on the fluorescence spectra of a set of blood plasma biomolecules [tyrosine, tryptophan, nicotinamide adenine dinucleotide (NAD), and flavin adenine dinucleotide (FAD)] and red blood cell (RBC)-associated porphyrin is being evolved by our group.

**Results:**

The research so far has exhibited sensitivity and specificity values exceeding 90% based on the spectral features of blood components of 14 malaria patients and 20 numbers of age adjusted normal controls. The fluorescent biomolecules go out of proportion when the malarial parasite breaks down the hemoglobin of blood.

**Conclusion:**

This technique has the potential to be used as an alternative diagnostic procedure for malaria since the instrumentation involved is portable and inexpensive.

**Virtual Slides:**

The virtual slide(s) for this article can be found here: http://www.diagnosticpathology.diagnomx.eu/vs/13000_2014_182

## Background

Malaria is considered as one of the major health problems in subtropical countries like India and Bangladesh. It is a mosquito borne infectious disease, but caused by the parasite of the genus Plasmodium. The disease is transmitted through the bite of mosquito infected by the parasite. The mosquito bite introduces the malarial parasite from its saliva into the blood of the human host. The parasite travels to the liver to reproduce and mature up [1]. The common symptoms due to malaria include headache and fever, shivering, joint pain, convulsions etc., [2]. Malaria is a curable disease, if treated in earlier stages, but delay in treatment might result in death. The most common medications used for treating malaria include quinone (chloroquinone), artemisinin derivatives, in combination with mefloquine. Malaria is prevalent in the rainy season as the stagnant waters provide an adequate environment for the development of mosquito larvae. Since, there are no effective vaccines for malaria, the transmission of disease can be prevented by avoiding mosquito bites using repellents, nets and also applying insecticides on stagnant waters. [3].

When the sporozoites of the malaria parasites enter into the bloodstream through a mosquito bite, they migrate to the liver. The sporozoites infect the liver hepatocytes and get multiplied into merozoites asexually inside the hepatocytes (8–30 days). After maturing, they break the cell walls of hepatocytes and enter into the blood stream. Like the sporozoites, which multiplied inside the hepatocytes, the merozoites infect RBCs, get multiplied further within, finally breaking out of RBCs to invade fresh RBCs [4].

In humans, malaria is caused by five species of Plasmodium including Plasmodium falciparum, Plasmodium vivax, Plasmodium malariae, Plasmodium ovale and Plasmodium knowlesi. The three major human malaria species in India are Plasmodium falciparum, Plasmodium vivax and Plasmodium malariae [5].

Plasmodium falciparum is the most dangerous form of malaria with the highest rate of mortality. The treatment for malaria will be provided on the basis of the species identified upon standard diagnosis. In the states where Plasmodium falciparum and Plasmodium vivax co-exist, fluctuating proportions of the two species complicate the diagnosis and treatment process [5].

The diversity of malaria in India is also attributed through the increased diversity of malaria vector species. In India, of the 60 species of mosquito available, six were found to be the vector for malaria. Anopheles culicifacies with its indistinguishable sibling species: A, B, C, D, and, E is the major vector of malaria in India which accounts for 60 - 65% of the malaria burden. It also acts as a model organism to study the host-vector-parasite interactions [6].

Microscopy of stained thick and thin blood smears helps in the diagnosis of malaria. Their advantages include ease of use, inexpensiveness; but the major disadvantage is the moderate sensitivity (56-70%) [7]. Rapid Diagnostic Tests such as antigen-antibody enzymatic analyses were developed to detect the circulating parasites and their antigens [8]. An advanced technique is available, that uses the PCR to detect the parasite’s DNA, but is not widely used due to the high cost and complexity [6]. Some preliminary works have been done by others, employing spectroscopic techniques, based on UV- VIS absorption for monitoring malarial diseases [9,10].

Spectral diagnosis is a newly evolving technique for detecting diseases, particularly cancer [11-16], thalassemia and sickle cell anemia [17]. This method is based on the difference in the concentration of fluorescent bio molecules, which indirectly acts as biomarkers for each type of disease. In continuation of our earlier works in this line, the paper presented here is a preliminary investigation to explore the possibility of diagnosing malaria by the spectral features of the infected blood.

## Methods

The methods and materials for spectral diagnosis of cancer and hemoglobinopathies have been presented in a number of papers earlier [11-19]. The same procedure had been adopted here too. The spectral features of blood components [plasma and acetone extract of RBC] were obtained from 14 persons [male 8, female 6 with age ranging from 10 – 20] diagnosed with malaria. In order to highlight the distinct differences, the spectral features of samples of normal control [N = 20] have been presented and compared.

### Blood

Controls: Exactly 5 ml of venous blood from each of the 20 healthy volunteers (age range: 10 to 20 years) was collected in a violet sterile vial that contained the anticoagulant EDTA. The vial was gently rocked five times to adequately mix the EDTA and whole blood, and the samples was centrifuged at 3000 rotational speed (RPM) for 15 minutes. Clear, pale, greenish-yellow plasma supernatant was obtained by such centrifugation. A total of 1.5 ml of supernatant was removed from the top layer for spectrofluorimetric analysis, leaving the buffy coat and the formed elements, as undisturbed sediment. The blood plasma sample was subjected to synchronous fluorescence excitation spectral analyses without any other treatment.

Next, the buffy coat, which contained mostly white blood cells (WBCs) (e.g., lymphocytes), was removed and discarded, and exactly 1 ml of the thick formed elements from the bottom layer, which contained mostly RBCs, was removed to a sterile vial and mixed with 2 ml of analytical grade acetone. Proper care was taken to ensure that the formed elements did not develop lumps. After thorough mixing to enable the acetone to extract fluorophores within and around the cells, the sample was centrifuged again (3000 rpm for 15 minutes). The resulting supernatant was subjected to fluorescence emission spectra analysis at an excitation wavelength of 400 nm.

Patients: The same protocol was used to process blood samples from confirmed patients suffering from malaria caused by the parasite plasmodium falciparum as diagnosed by professional pathologists of General hospital, Chennai. Samples were obtained before any treatment. The patients were informed about the investigation, and proper consents were obtained. The samples were collected as per the ethical committee approval (GACN/KP/CNBT/8428) The malaria subjects consisted of 14 patients (male 8, female 6 with age ranging from 10 – 20), and had different levels of clinical severity.

### Instrumentation

The instrument used was a spectrofluorometer (SL 174, Elico, India) capable of collecting excitation, emission, and synchronous spectra in the 200–800 nm range. An excitation and emission slit width of 10 nm and scan speeds of 1000 nm/min were used. The samples were placed in quartz cuvettes and illuminated by a specified wavelength of light with a 10-nm spectral width and a spot size of 3 × 2 mm. The power at the point of illumination was approximately 20 μW, which was too low to induce photo bleaching. This finding was confirmed by repeating the experiment three times for each sample and observing no inter-replicate spectral differences.

Three types of spectra are measured in the field of fluorescence spectroscopy. In fluorescence emission spectra (FES), one particular wavelength is selected for the excitation of a molecule, and the fluorescence emission spectrum is obtained by rotating the emission grating over a predetermined range. The reverse is true of fluorescence excitation spectra (FXS), in which the peak emission band of a molecule is selected, and the excitation grating is rotated to scan the excitation spectra. In synchronous excitation spectra (SXS), both gratings are synchronously rotated at offsets of 40 nm or 70 nm to obtain the fluorescence excitation bands for every molecule in the predetermined range. The wavelength offset and scan range are not unique; they are determined empirically by trial and error, for a given set of experimental protocol. After analysing other offsets, including 10 nm and 30 nm, it was determined that the 70 nm offset provided excellent resolution and good contrast between the normal and malaria samples because 70 nm is the Stokes shift [20] of the most important biofluorophores (e.g., tryptophan, nicotinamide adenine dinucleotide etc.) in blood plasma. Hence, all of the results presented for plasma were based on the synchronous excitation spectra (SXS). The blood plasma is a complex matrix, with many biofluorophores with overlapping absorption and emission bands. The biggest advantage of synchronous scan, with ∆70 nm offset, is to limit the spectra with three distinct bands.

It is important to draw attention to the fact that concentration quenching and inner filter effect are inherent in such undiluted plasma. Hence the experiments were repeated for both sets of samples, after 5 times and 10 times serial dilution with normal saline. However, discrimination between the samples of patients and controls were best with undiluted samples and hence only this result was presented.

## Results

**Figure 1:**
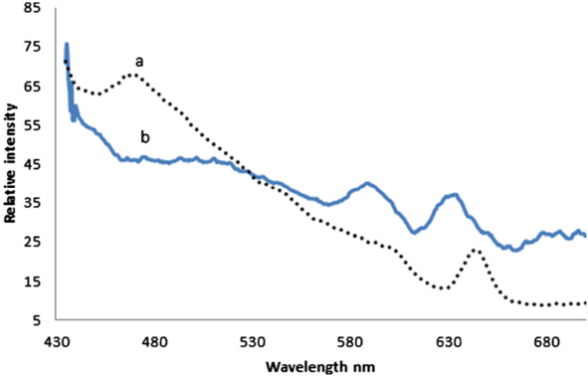
**Fluorescence emission spectra (FES) of acetone extract of formed elements of (a) Normal control (b) Malaria patient.**

Figure 1 (a), represents the typical fluorescence emission spectra (FES) of acetone extract of RBC of normal control (male, age 12). This spectra was obtained with the excitation wavelength fixed at 400 nm and emission scanned from 425 to 675 nm.

It could be seen that there were three important fluorescence bands, 460 nm (due to the metabolite flavin adenine dinucleotide, FAD), 585 (due to basic from of porphyrin, a protein found in hemoglobin), 635 nm (due to the neutral form of porphyrin). In fluorescence spectroscopy, the accepted convention is to take the ratio of peaks of fluorescence bands, in order to quantity and obtain a measure of endogenous biofluorophores. If we define the ratio R_1_ = I_635_/I_585,_ the ratio of intensity of two fluorescence bands cited above, this was about 1.2 ± 0.1 for normal control.

Figure 1 (b), represented the typical fluorescence spectra of acetone extract of RBC of a malaria patient (male age 18). The spectral features were similar to those of normal control, with the ratio R_1_ = 1.0 ± 0.1. That means, as far as porphyrin is taken as the biomarker, the difference between the malarial sample and normal control sample is only marginal. (The hump at 500 nm, in the above spectrum was again due to FAD and this shift was not consistent and hence ignored).

**Figure 2:**
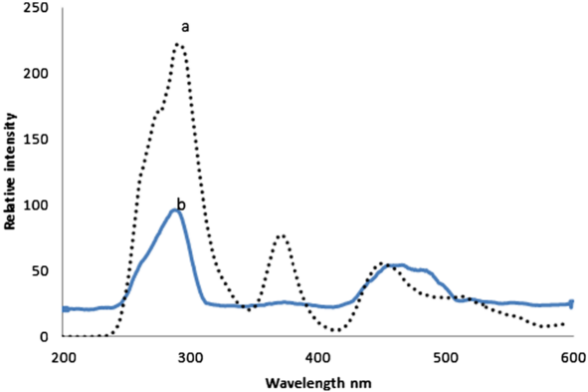
**Synchronous excitation spectra (SXS) of blood plasma of (a) Normal Control (b) Malaria patient.**

Figure 2 (a) represented the SXS, the synchronous excitation spectra of normal blood plasma. It showed three main bands; at 280 nm (due to the excitation peak of the amino acid tryptophan); at 380 nm (due to co enzyme Nicotinamide adenine Dinucleotide, NADH); and at 460 nm (due to metabolite FAD). A ratio parameter R_2_ = I_280_/I_380_, (ratio of peak intensity at 280 nm and 380 nm) was 4 for normal; another ratio parameter R_3_ = I_460_/I_380_ (ratio of peak intensity at 460 nm and 380 nm) was about 1.0 for normal.

Figure 2(b) showed the SXS of blood plasma of the above malaria patient. This showed three main bands at 280 nm, 380 nm and 460 nm, (like those of normal plasma), but these bands were markedly out of proportion. For example, R_2_ = 6.9, R_3_ = 3.2 for the malarial sample. That is, R_2_ was about 70% and R_3_ was 300% elevated respectively.

**Figure 3:**
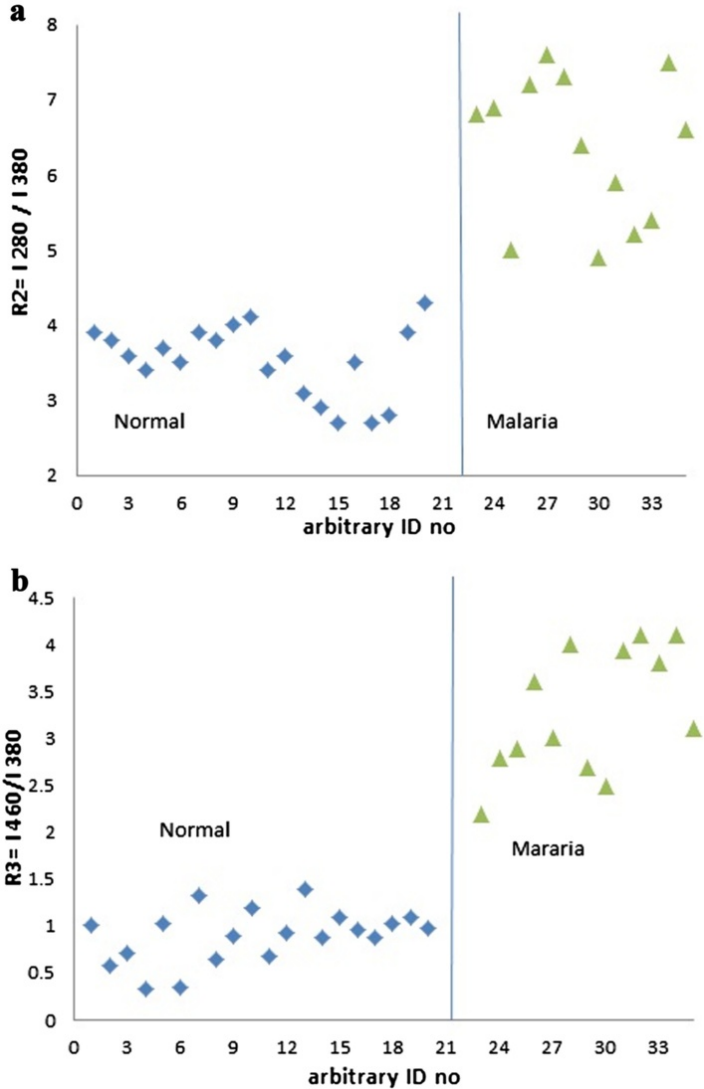
**Discrimination between malaria and control samples. a)** Scatter plot of R2 values (I 280/I380),obtained from SXS of plasma **(b)** Scatter plot of R3values (I460/I380), obtained from SXS of plasma.

Figure 3 (a) represented the scatter plot for R_2_ and Figure 3 (b) for R_3_ to classify the malaria samples from the control ones. The discrimination between the two sets was quite good.

## Discussion

Spectral diagnosis is a newly evolving technique for detecting diseases, particularly cancer [11-16], thalassemia and sickle cell anemia [17]. It is based on the difference in the concentration of fluorescent biomolecules, which indirectly act as biomarkers. The same technique has been extended to the detection of malaria to explore the diagnostic potential since the instrumentation employed here was simple and inexpensive.

Out of the few diseases diagnosed by spectral features, it was thalassemia closest to the malaria, in terms of spectral manifestation. Thalassemia are inherited diseases due to reduced or defective α or β chain of the hemoglobin (Hb). Because of this, the concentration of RBC is only about 50 to 60% of the normal [17]. For example, the Hb level is 6 g/dL for thalassemia patients; but for men, 13.5 to 17.5 g/dl, for women 12.0 to 15.5 g/dl for normal controls. Such a decrease in RBC has manifested correspondingly in R_1_ as shown in our earlier papers [17]. In comparison, for malaria patients, R_1_ has decreased only marginally or from the window of R_1_ the sensitivity and specificity of malaria diagnosis would be very poor.

Another important aspect of thalassemia is the poor average lifetime of RBC, while the average lifetime for normal controls is about 120 days for the thalassemia patients it varied from 80 days to 10 days depending upon the severity. This is because the RBC of thalassemia patients is fragile. This is manifested as the abnormally high value of R_2_ and R_3_ as these two parameters area measure of metabolites of hemoglobin [17-19]. For example, NADH is abnormally low and FAD is high for thalassemia samples, because of which R_2_ and R_3_ is elevated. When RBC breaks down, a process that is abnormally high in thalassemia patients, the consumption of NADH is high, simultaneously producing equally abnormally high levels of FAD.

Similar degradation is externally initiated and perpetrated by the parasite which breeds and multiplies inside the RBC of malaria infected patients. In other words, malaria and thalassemia are similar from the point of view of the fragility of RBC the former being initiated by the parasite injection, and the latter being inherited. However, these two are dissimilar from the point of concentration of RBC as the former ones are persons with the same concentration of RBC as those of normal, but the latter ones are subjected with inherited low level of RBC.

**Figure 4:**
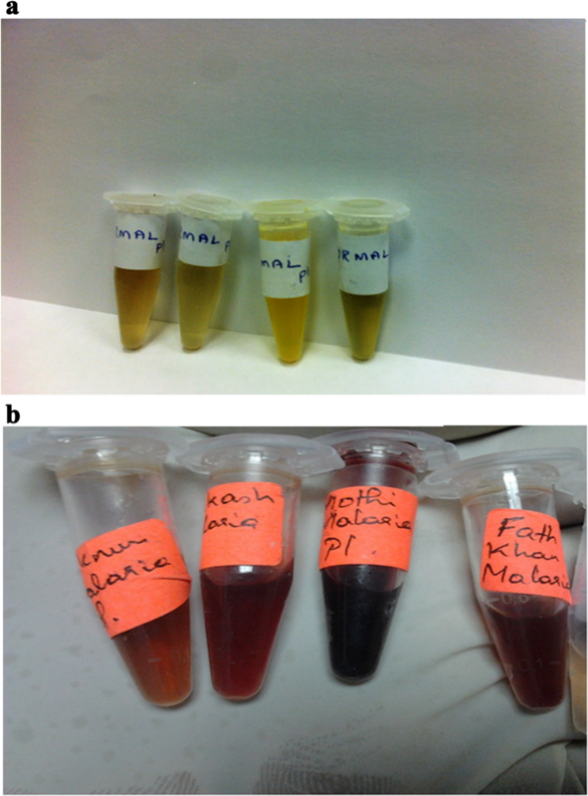
**Photographs of blood plasma samples (a) of normal control (b) of malaria patient.**

Most of the symptoms and complications of malaria may be attributed to the rapid and abnormal lysis of the RBC’s and this spectral diagnosis provide the direct evidence for this point of view. The photographs, presented in Figure 4 (a) for the plasma of normal and 4 (b) for patient, show the distinct difference. The pale reddish plasma of malarial patient is due to degradation of RBC induced by the parasite. In contrast the control plasma was pale greenish yellow.

## Conclusion

In this preliminary study, done as a proof of concept, the fluorescence spectral features of blood components of malarial patients have been reported. The study shows that, the malarial patient sample could be distinguished with high sensitivity from the normal control, because plasma of a malaria patient is flooded with the fluorescent decay products of RBC. It is important to mention that this report presents results of only one type of malaria (PF). We need to repeat with other types of malaria to check possible distinction, a task difficult in the conventional technique. In addition, we need to do large-scale, multicenter study to assess the clinical viability of this new technique. We hope to undertake this task in the near future.
